# Perineal wound disruption and postoperative infection following resection of a benign cystic sacrococcygeal teratoma in a neonate in a resource‐limited setting: A case report

**DOI:** 10.1002/ccr3.2124

**Published:** 2019-04-02

**Authors:** Christine Katusiime

**Affiliations:** ^1^ Luvley Medical Institute Kampala Uganda

**Keywords:** case report, perineal wound disruption, postoperative infection, resource‐limited settings, sacrococcygeal teratomas

## Abstract

Case reports highlighting the complications of SCT surgical resection in resource‐limited settings particularly sub‐Saharan Africa are few. It is imperative to take into account that achieving the desirable cosmetic results may not be possible because of large tumor size and postoperative infection. It is therefore of necessity to consider integration of plastic surgical reconstructive programs into pediatric surgery follow‐up programs in resource‐limited settings.

## 1 INTRODUCTION

SCTs are rare in occurrence with an incidence of 1 in 35,000 to 40,000 live births predominantly occurring in females in comparison with males in the ratios of 4:1, respectively.^1^, ^2^ Although there is a plethora of literature on SCTs, there are few case reports from sub‐Saharan Africa particularly describing complications of SCT surgery. To our knowledge, pediatric SCT surgical resection cases inclusive of complications in sub‐Saharan Africa have rarely been reported, and to this effect, we report a case of a neonate that developed postsurgical complications in a resource‐limited setting.

## 2 CASE REPORT

A 37‐year‐old P_4 + 0 _delivered a live female baby at the obstetrics unit of our hospital via normal spontaneous vaginal delivery. Antenatal care examinations during the 1st and 2nd trimesters were unremarkable. A routine obstetric ultrasound scan at 32 weeks of gestation, however, had confirmed the findings of a live intrauterine fetus with a sacrococcygeal mass.

The total weight gained during pregnancy was 15kg, and she was maintained on supplemental iron and folic acid.

At birth, the female newborn had a birth weight of 3.800kg. Apgar score was 9/10 and 10/10 in the first and fifth minutes, respectively. There were no significant findings on systemic examination.

**Figure 1:**
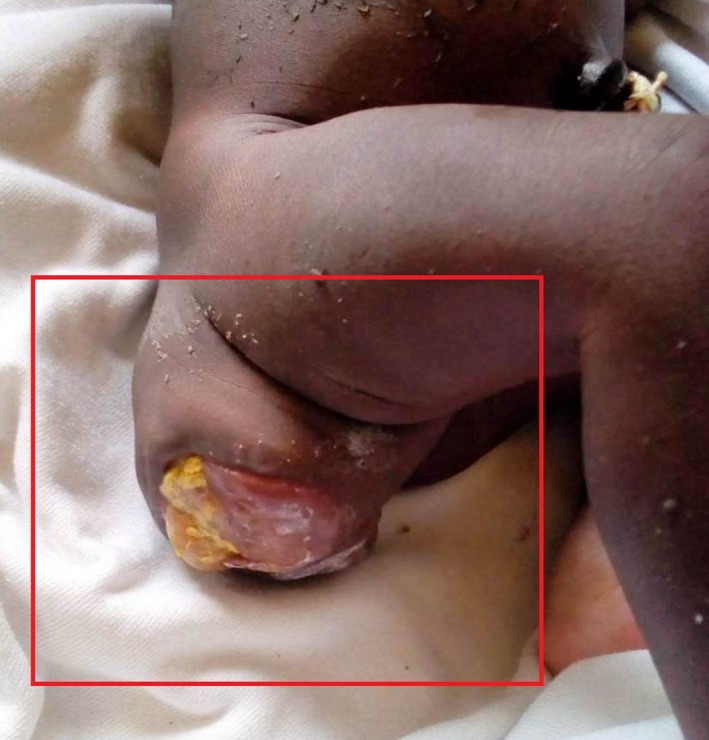
Image of pelvis showing sacrococcygeal teratoma in neonate

**Figure 2:**
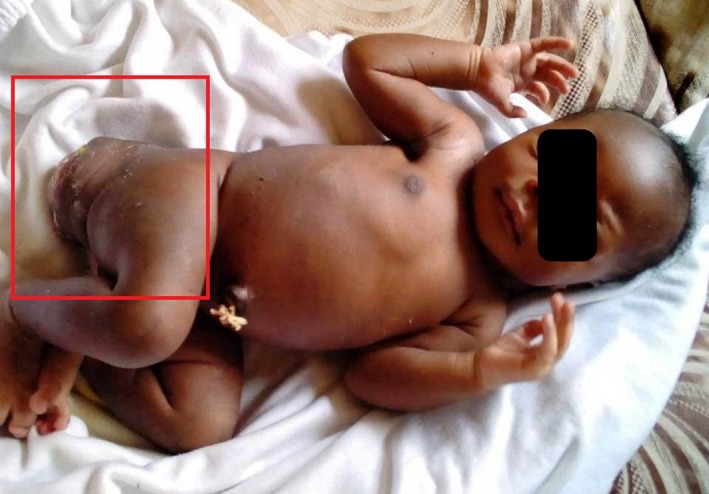
Image of sacrococcygeal teratoma in comparison with neonate

Local examination revealed a solid‐cystic, firm mass measuring 10cmx7cm in the sacral region with deviation of the anal orifice posteriorly (Figures 1 and 2).

Plain radiographs and abdominal ultrasound scans confirmed a sacrococcygeal tumor stage I Altman classification arising from the coccyx. There was no bladder, genitalia, or bowel involvement. The echocardiogram, electrocardiogram, and brain ultrasound were normal. Abdominal CT and MRI scans were not possible due to financial constraints.

Postnatal examination of the P_4 + 0 _was unremarkable.

All preoperative investigations on the newborn were essentially normal: Hb level 18g/dl, random blood sugar 105 mg/dl, serum alkaline phosphatase 110 IU/dl, PT 15, INR 1, BUN 30 mg/dl, hematocrit 33%, platelet counts 300000/l, and bilirubin 1.3 mg/dl. Cryptococcal antigen and syphilis serology (Venereal Disease Research Laboratory and *Treponema pallidum *hemagglutination assay) were negative.

**Figure 3:**
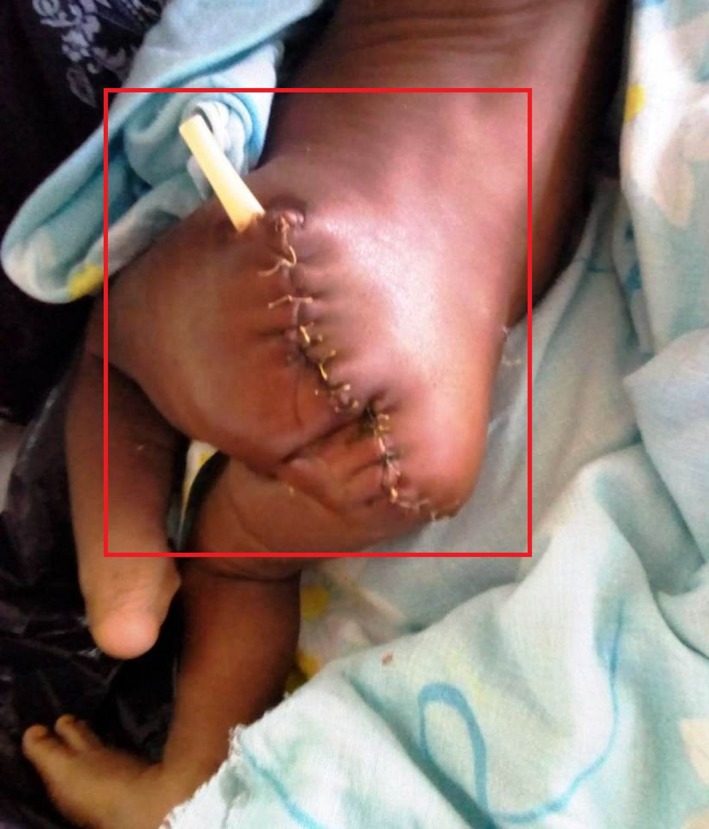
Initial postoperative sutures with drain in situ

Alpha‐fetoprotein (AFP) serological measurements were unable to be done due to financial constraints. Due to the unavailability of AFP titers, a decision of surgical excision was made. Tumor resection and coccygectomy were done 10 days after birth using the posterior sacral approach under general anesthesia (Figure 3).

An ulcerated cystic mass measuring 11cmX8cmX4.5cm with an ulcer measuring 4cmX3cm and weighing 175grams was resected. The mass contained cystic fluid and purulent material. Histopathological examination of the excised tissue revealed a multilocular cystic mature sacrococcygeal tumor with no evidence of malignancy. Immunohistochemical stains for AFP were positive.

**Figure 4:**
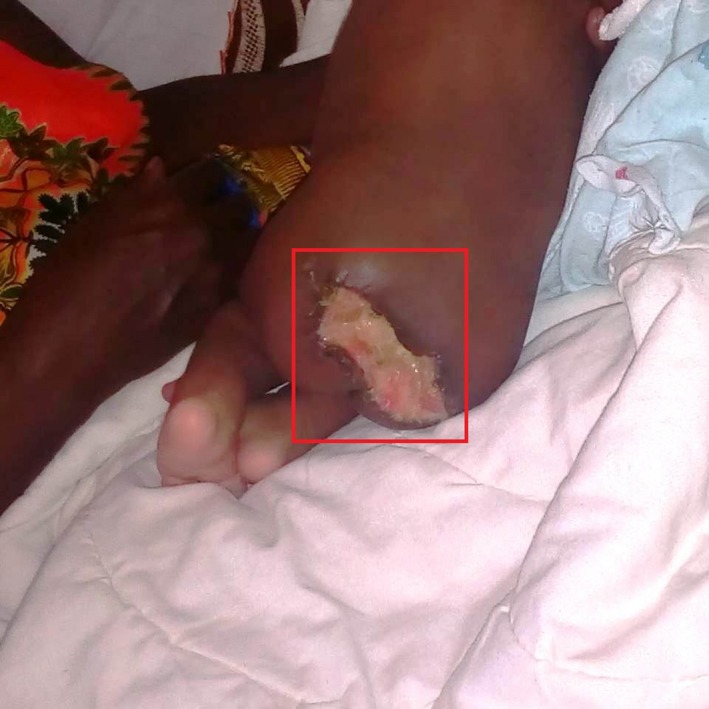
Perineal wound disruption and postoperative infection

On postoperative day 2, the stitches at the wound site gave way and the wound became septic (Figure 4). Blood cultures and wound site swabs were unremarkable. The baby was diagnosed with a surgical site infection.

Since the baby did not have symptoms of hyperbilirubinemia, a decision was made to manage the baby with postoperative intravenous antibiotics—a first‐generation cephalosporin—ceftriaxone 100mg/kg, a broad‐spectrum antibiotic—metronidazole 35mg/kg, paracetamol 7.5mg/kg after conducting debridement, irrigation with warm normal saline solution and refashioning in the operating theater.

The baby was managed with daily wound pressure bandage dressings in addition to saline wound cleaning. Baby was discharged on postoperative day 25.

**Figure 5:**
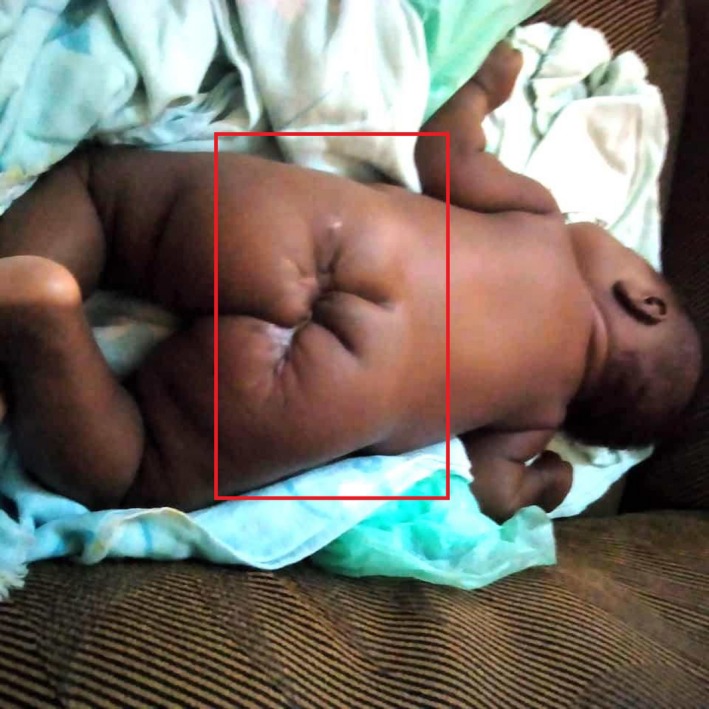
Poor cosmetic scar

Follow‐up at 6 months revealed that the child had a well‐healed though poor cosmetic scar (Figure 5) with no biochemical or physical evidence of recurrence. The child had achieved the age‐appropriate developmental milestones.

## 3 DISCUSSION

SCTs are congenital neoplasms that arise from Hansen's node or primitive germ cells and constitute tissue from all the three embryonic germ layers—endoderm, ectoderm, and mesoderm.^1^, ^2^

Although there is a plethora of literature on SCTs, there are few case reports from developing countries particularly sub‐Saharan Africa because neonatal surgery is of low priority in these settings.^4^ In Uganda, only 3.5% of the need for neonatal surgery is met by the health system.^5^

Whereas other congenital anomalies, for instance, sacral bone defects, spina bifida, spinal dysraphism, sacral agenesis, imperforate anus, presacral masses, scimitar sacrums, meningomyeloceles, meningoceles, pulmonary stenosis, ventricular septal defect, patent ductus arteriosus, ectopic kidney, anorectal malformations, duplication of the uterus or vagina, clubfoot, polydactyly, and anorectal malformations, are associated in 5 to 26% of patients with SCTs,^6^, ^7^ our patient did not have any of these anomalies.

The ideal diagnosis of SCTs in neonates is by obstetric ultrasound scanning as was in our patient which not only allowed for delivery planning and early neonatal surgery but also determined the overall prognosis of our patient.

Early diagnosis and surgical intervention also reduce the risk of malignant transformation seeing that less than 8% of SCTs at birth are malignant.^3^, ^9^, ^10^ There is evidence that suggests that preemptive early delivery by elective cesarian section when the tumor exceeds 5cm in diameter will avert teratoma rupture and hemorrhage.^11^

Although the possibility of cesarian section was discussed with our patient, she declined elective surgery.

Early surgical resection with coccygectomy as was in our patient is the primary treatment of SCTs. This not only carries a better prognosis but also is essential in ensuring a 30%‐40% reduction in tumor resurgence.^3^

Like any other surgical procedure, complications arising from surgical resection can either be short‐term—postoperative wound infection and perineal wound disruption (Figure 4) as was in our patient, rectal injury, diarrhea, tumor rupture, hemorrhage, septicemia, or be long‐term—urethral obstruction, midurethral necrosis, anorectal dysfunction, fecal incontinence, severe chronic constipation, neurourological dysfunction of the lower urinary tract, bladder sphincter dysfunction, vesicoureteral reflux, urinary incontinence, neurogenic voiding dysfunction, mixed neurogenic damage, abnormal bladder, and urethral functions diminished sexual function in females, deformity, unpleasant scars, and tumor recurrence.^8^, ^12^, ^13^

This case report also demonstrates the dilemma of poor cosmesis (Figure 5) following neonatal surgery which was due to two factors: a large tumor and postoperative wound infection and the necessity of integration of plastic surgical reconstruction into pediatric surgery follow‐up programs.

## 4 CONCLUSION

Case reports highlighting the complications of SCT surgical resection in resource‐limited settings particularly sub‐Saharan Africa are few. It is imperative to take into account that achieving the desirable cosmetic results may not be possible because of large tumor size and postoperative infection. It is therefore of necessity to consider integration of plastic surgical reconstructive programs into pediatric surgery follow‐up programs in resource‐limited settings.

## CONFLICT OF INTEREST

None declared.

Consent of Ethics: Not applicable.

Informed Consent: Obtained.

## AUTHOR CONTRIBUTION

CK: is the corresponding author; clinician; involved in the study, design, and analysis of data; and drafted and critically revised the manuscript.
